# Complement split product C3c in saliva as biomarker for periodontitis and response to periodontal treatment

**DOI:** 10.1111/jre.12788

**Published:** 2020-07-18

**Authors:** Maria Anastasia Grande, Daniel Belstrøm, Christian Damgaard, Palle Holmstrup, Sai Sindhu Thangaraj, Claus Henrik Nielsen, Yaseelan Palarasah

**Affiliations:** ^1^ Section Clinical Oral Microbiology, Periodontology Faculty of Health and Medical Sciences Department of Odontology University of Copenhagen Copenhagen Denmark; ^2^ Section for Oral Biology and Immunopathology, Periodontology Faculty of Health and Medical Sciences Department of Odontology University of Copenhagen Copenhagen Denmark; ^3^ Institute for Inflammation Research Center for Rheumatology and Spine Diseases Rigshospitalet Copenhagen University Hospital Glostrup Denmark; ^4^ Faculty of Health and Medical Sciences Department of Cancer and Inflammation Institute of Molecular Medicine University of Southern Denmark Odense Denmark

**Keywords:** complement component 3, intervention, periodontitis, saliva

## Abstract

**Background and Objective:**

The complement system is engaged in inflammatory reactions both in the periodontal pockets and in the periodontium itself, where it can mediate tissue destruction. The aim of this study was, first, to compare salivary levels of the total complement system protein C3 and its split product, fluid‐phase C3c in patients with periodontitis and periodontally healthy controls. Next, to determine if C3 and C3c levels had biomarker potential in diagnosing and monitoring periodontitis and its treatment. We hypothesized that salivary levels of total C3 and the split product C3c associated with the severity of periodontitis and reflected decreased inflammatory activity after periodontal treatment.

**Methods:**

At baseline, stimulated saliva samples were collected from patients with periodontitis (n = 18) and periodontally healthy controls (n = 15). Subsequently, non‐surgical periodontal treatment was performed in the patients, and saliva sampling from patients was repeated two‐, six‐, and twelve weeks post‐treatment (NCT02913248 at clinicaltrials.gov).

The patients were grouped as good and poor responders to treatment according to the achieved reduction in bleeding on probing (BOP).

Salivary levels of C3 and C3c were quantified using sandwich ELISA.

**Results:**

Patients with periodontitis had higher baseline levels of both total C3 and the split product C3c in saliva than did periodontally healthy controls (*P* < .0001). Receiver operating curve (ROC) analyses discriminated patients with periodontitis from controls based on both C3 (AUC (area under curve) = 0.91, *P* < .001) and C3c levels (AUC = 0.84, *P* < .001) in saliva. Periodontal treatment improved all clinical parameters (*P* < .01). Good responders (n = 10) had lower baseline levels of C3c than poor responders (n = 8), (*P* < .05), and baseline levels of C3c discriminated between good and poor responders (AUC = 0.80, *P* < .05).

**Conclusion:**

In conclusion, patients with periodontitis had higher salivary levels of C3c, and the C3c levels were predictive of reductions in BOP, that is, the poor responders. This suggests that salivary C3c levels possess potential to serve as a biomarker predicting the clinical response to non‐surgical periodontal treatment.

## 1 INTRODUCTION

Periodontitis is a multifactorial inflammatory disease in the tooth‐supporting tissues, which affects nearly 50% of the adult population in the Western world.^1^ Periodontitis is initiated and maintained by a subgingival and polymicrobial biofilm, which in susceptible individuals leads to destructive inflammation.^2^ Ultimately, periodontitis can cause tooth loss and simultaneously occurrence of systemic diseases such as cardiovascular disease, diabetes, and rheumatoid arthritis.^3^, ^4^, ^5^ Therefore, early diagnostic and treatment are essential.

The complement system consists of more than 40 proteins and plays an important role in the combat of microorganisms, recruitment and regulation of inflammatory cells, and in clearance of apoptotic host cells and immune complexes.^6^ In periodontitis, bacteria modulate and activate the complement system, the latter resulting in cleavage of complement component C3 into C3a and C3b.^7^, ^8^, ^9^ C3b becomes covalently attached to the bacterial surface and is enzymatically converted into iC3b. Subsequently, iC3b is cleaved into fluid‐phase C3c and surface‐bound C3dg.^7^, ^10^ C3dg and C3c may thus serve as markers of ongoing formation and degradation of the biologically active C3 fragments C3b and iC3b.

While measurement of C3 levels in blood is used to screen for specific inflammatory medical diseases such as systemic lupus erythematosus,^11^, ^12^ the inflammatory oral disease periodontitis is diagnosed based on clinical and radiographic recordings.^13^ These recordings detect manifest tissue changes, which are indicative of treatment needs, but their value in diagnosing periodontitis at early disease stages and in predicting treatment responses are limited. Screenings for complement proteins and their split products in saliva may offer earlier diagnoses and personalized treatment plans, as the content of complement proteins and their split products in saliva may reflect local production and extravasation of complement proteins, as well as their activation locally.^14^, ^15^

Previous investigations have shown associations of complement protein levels with periodontal inflammation^6^, ^15^, ^16^, ^17^, ^18^, ^19^, ^20^; however, the C3c‐specificity of the assays previously used is limited. Many commercially available C3c‐antibodies recognize both total C3, hydrolyzed C3 (C3_(H2O)_) and degradation fragments containing the C3c‐moiety (C3b, iC3b).^21^ We have raised a monoclonal antibody that only recognizes a neo‐epitope in C3c, which becomes accessible when iC3b is cleaved into fluid‐phase C3c and bound C3dg.^22^ Consequently, our assay detects C3c detached from C3dg, which reflects local complement consumption and potentially also the ongoing inflammation due to a short half‐life of C3c.^23^

The aim of this study was, first, to determine the value of salivary C3 and C3c as diagnostic biomarkers for periodontitis and, secondly, to determine if C3 and C3c levels had biomarker potential in monitoring periodontal treatment. We hypothesized that salivary levels of total C3 and the split product C3c associated with the severity of periodontitis and reflected decreased inflammatory activity after periodontal treatment.

## 2 METHODS

### 2.1 Study population

This study is part of a series of investigations analyzing salivary biomarkers in patients with periodontitis receiving non‐surgical periodontal treatment.^24^, ^25^ The clinical trial itself was performed in 2017, which is why patients with periodontitis were enrolled based on definitions from American Academy of Periodontology 2015.^26^ However, when incorporating the new classification of periodontitis, all patients were categorized in stage two or three (moderate to severe periodontitis).^27^ Patients with generalized moderate to severe periodontitis (n = 25) and periodontally healthy controls (n = 15) completed the study. All patients fulfilled the inclusion criteria: ≥ 4 teeth with periodontitis (BOP, PD ≥ 5 mm, CAL ≥ 3 mm, radiographic bone loss ≥ 3 mm^26^), age ≥ 40 years., teeth ≥ 20, Caucasian, and exclusion criteria: treatment‐requiring caries, hyposalivation, systemic diseases and current use of medication with known effect on the periodontium, professional dental cleaning and/or antibiotic treatment within the latest three months.

The periodontally healthy controls were all dental students from the University of Copenhagen. They were enrolled based on the criteria: teeth ≥ 20, no presence of periodontitis, gingivitis, or treatment‐requiring caries, no systemic diseases or current use of medication with known effect on the periodontium. Before commissioning, all participants gave their written informed consent.

The study is accepted by the regional ethical committee of the capital region of Denmark (H‐16016368), reported to the Danish Data Authority (SUND‐2016‐58) and registered at clinicaltrials.gov (NCT02913248).

### 2.2 Study design

The present study is an interventional study with a follow‐up period of three months, which has previously been described in detail.^24^, ^25^ In brief, baseline paraffin‐stimulated saliva samples were collected from patients with periodontitis and periodontally healthy controls.

Subsequently, in patients with periodontitis, full‐mouth periodontal recordings (third molars excluded) of plaque index (PI), bleeding on probing (BOP), probing depth (PD), and clinical attachment level (CAL) were measured at six sites per tooth. Then, full‐mouth non‐surgical periodontal treatment was performed including oral hygiene instructions, scaling, and root planing. No antibiotic treatment was administered. Follow‐up visits and periodontal recordings were performed two (PI and BOP), six (PI and BOP), and twelve weeks (PI, BOP, PD, and CAL) post‐treatment. Hygiene instructions were repeated, if plaque was visible upon application of erythrosine. Saliva was sampled at every visit before any clinical intervention and collected from 8 am to 3 pm Intra‐individual samplings were within a time interval of 4 hours. The samples were immediately frozen and stored at −80°C until further analysis.

Saliva samples from the periodontally healthy controls were collected in May 2018, and periodontal treatment progressed from September 2016 to the beginning of January 2017. Post‐treatment, the patients were grouped in good and poor responders based on their response to treatment, classified according to a reduction in BOP above or below mean. The same clinician (Maria Grande) performed all recordings, treatments, and samplings at the Department of Odontology, University of Copenhagen.

### 2.3 Analysis of complement factors

Established sandwich ELISAs were used to assess total C3 and split product C3c levels in saliva samples from patients and controls, as described.^22^ Maxisorp plates (Nunc) were coated with either 100 μL capture antibody mAb clone F1‐23 anti‐C3 total (2 μg/mL) or mAb clone F1‐4 anti‐C3c (6 μg/mL) and placed overnight at 4°C. The wells were then washed three times with washing buffer (Phosphate buffered saline (PBS) 0.05% Tween‐20), and saliva were added in a twofold dilution starting with a 1:10 dilution. The wells were incubated at room temperature for one hour, emptied and washed again before the next one‐hour incubation with either 100 μL of 2 μg/mL biotinylated polyclonal C3c/d (for total C3) or 100 μL of 2 μg/mL biotinylated polyclonal C3c. After another wash, 100 μL of streptavidin‐conjugated horseradish peroxidase diluted 1:5000 in PBS (Invitrogen) was added and incubated for 30 minutes. The microtiter plates were color developed in darkness for 20 minutes at room temperature by addition of 100 μL ortho‐phenylenediamine (0.5 mg/mL; Kem‐En‐Tec Diagnostics) dissolved in citrate buffer (35 mmol/L citric acid, 65 mmol/L Na2PO4 pH 5) containing 0.12‰ (vol/vol) H_2_O_2_. The color reaction was stopped by addition of 100 μL of 1 mol/L H_2_SO_4_ to each well. Optical density (OD) levels were measured at 490‐650 nm by using a V‐Max kinetic reader (Molecular Devices). The calibrator curve was made by a diluted EDTA plasma pool, and the quantification of total C3 and C3c was based on a 4‐parameter analysis of a fitted calibration curve using OD values obtained by serial dilutions of EDTA plasma pools (Software Softmax Pro^®^), (Molecular Devices).

### 2.4 Statistical analyses

All data were visually assessed for normal distribution using box‐plot illustrations and histograms. Differences between groups in total C3 and C3c concentrations were analyzed using Mann‐Whitney tests. Alterations in clinical parameters were analyzed using repeated measures ANOVA (PI and BOP) and paired‐samples t tests (Log10 transformed PD and CAL). Alterations in total C3 and C3c concentrations before and after treatment were analyzed using Friedman test. Area under curve (AUC) was used to discriminate between periodontal health and periodontitis, and between good and poor responders, both with respect to C3 and C3c values. A *P*‐value < .05 was considered statistically significant in all analyses. GraphPad Prism version 8 (GraphPad software) and SPSS‐statistics version 26, (IBM) have been used as statistical software.

## 3 RESULTS

Seven samples were excluded from the analyses, because of too low sample volume. This resulted in exclusion of all samples belonging to seven patients with periodontitis. Samples from the remaining 18 patients with periodontitis and 15 periodontally healthy controls were included in the study. In two samples, the values of C3c were below the levels of detection (LOD) and therefore substituted with the value, LOD/√2, for the statistical analyzes.^28^, ^29^

### 3.1 Salivary levels of C3 and C3c in patients with periodontitis and periodontally healthy controls

**Figure 1:**
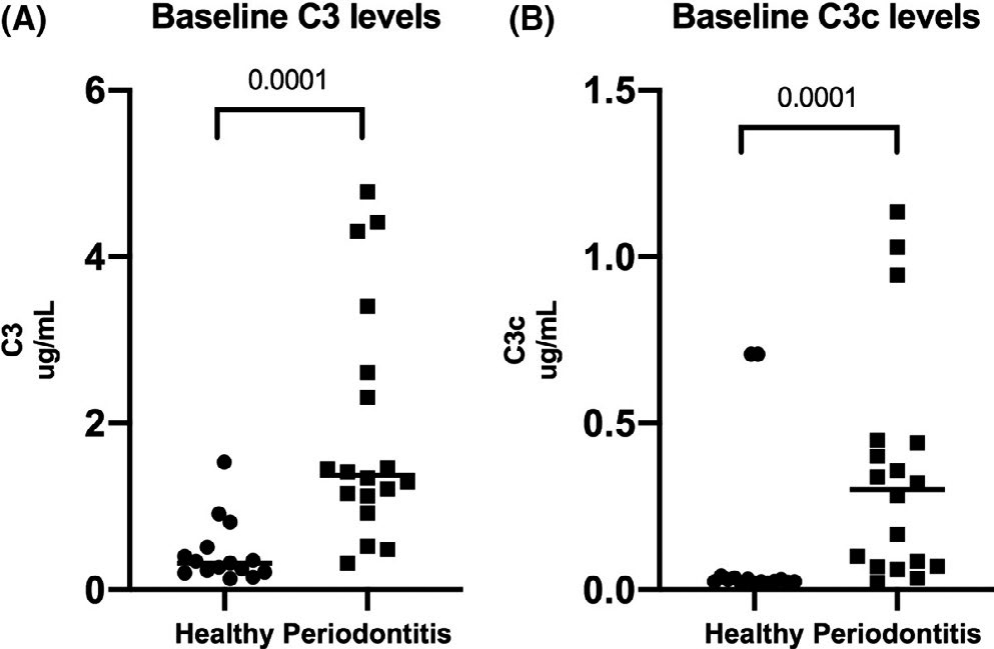
Baseline salivary levels of C3 and C3c in patients with periodontitis and periodontally healthy controls. The scatter dot‐plots illustrate salivary levels of (A) C3 (μg/mL) and (B) C3c (μg/mL) in patients with periodontitis (n = 18) and periodontally healthy controls (n = 15), (*P* < .0001). The horizontal lines show median levels

Significantly higher baseline levels of both C3 and C3c were observed in the patients with periodontitis, as compared to the periodontally healthy controls (*P* < .0001), (Figure 1).

**Figure 2:**
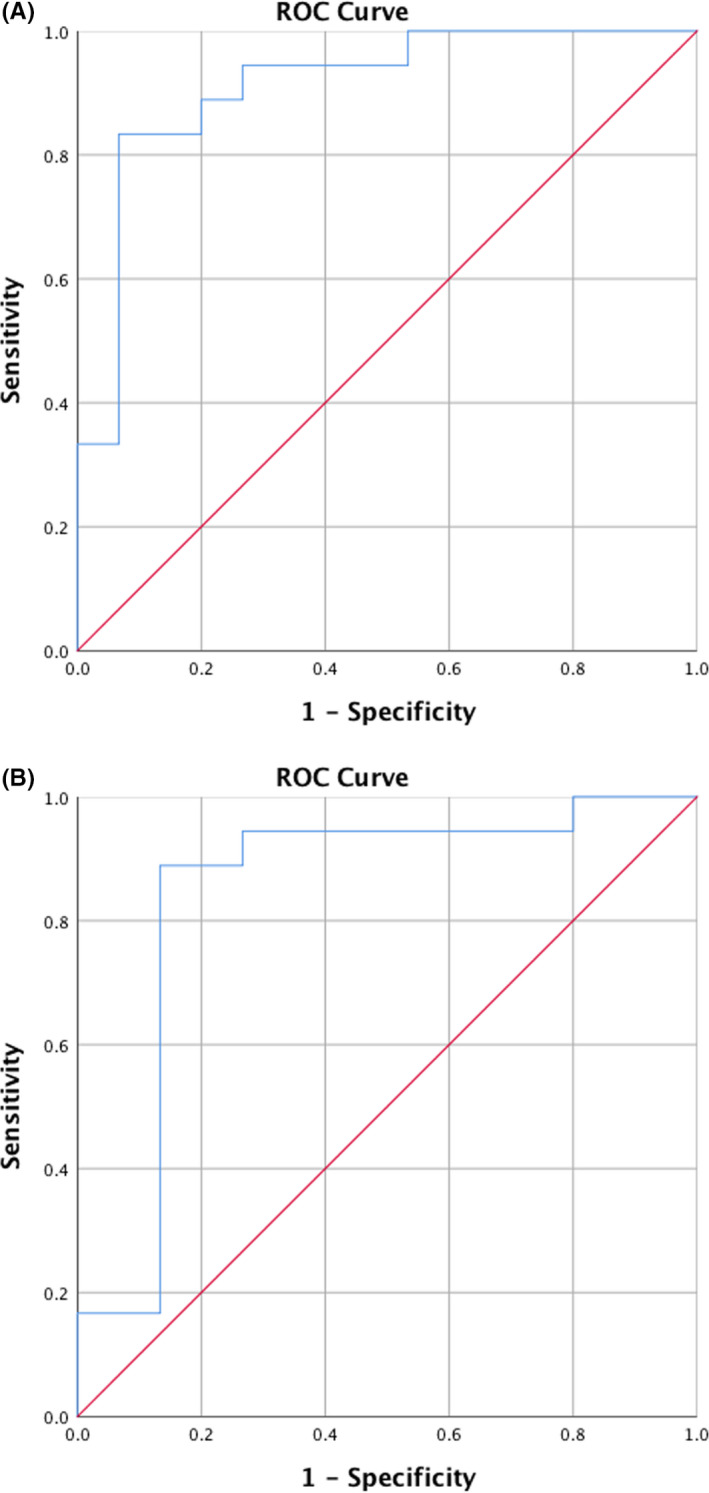
Area under curve (AUC) for C3 and C3c in identifying periodontitis from periodontal health at baseline. Receiver operating curves (ROC) of salivary A = C3 and B = C3c levels discriminating between patients with periodontitis and periodontally healthy controls at baseline. (A) C3, AUC = 0.91, CI = [0.81;1.00], (*P* < .001), (B) C3c, AUC = 0.84, CI = [0.69;1.00], (*P* < .001)

Using ROC analyses, high AUC values for C3 (AUC = 0.91, CI = [0.81;1.00]) and C3c (AUC = 0.84, CI = [0.69;1.00]) illustrated that levels of both markers in saliva were able to distinguish patients with periodontitis from periodontally healthy controls (*P* < .001), (Figure 2).

### 3.2 Effect of non‐surgical periodontal treatment on clinical parameters and salivary levels of C3 and C3c

The effect of the non‐surgical periodontal treatment on clinical parameters in this cohort has previously been described.^24^, ^25^ In short, the periodontal treatment improved all periodontal registrations of PI, BOP, PD, and CAL throughout the study period (Table 1).

**Table 1:** Clinical parameters before and after non‐surgical periodontal treatment in patients with periodontitis

	Baseline	Week 2	Week 6	Week 12	*P*‐value
Mean PI	85%	45%	43%	42%	<.001
Mean BOP	56%	31%	36%	44%	<.001
Mean PD	3.4 mm	ND	ND	3.0 mm	<.01
Mean CAL	4.1 mm	ND	ND	3.8 mm	<.01

Abbreviations: BOP, Bleeding on probing; CAL, Clinical attachment level; ND, Not determined; PD, Probing pocket depth; PI, Plaque index.

**Figure 3:**
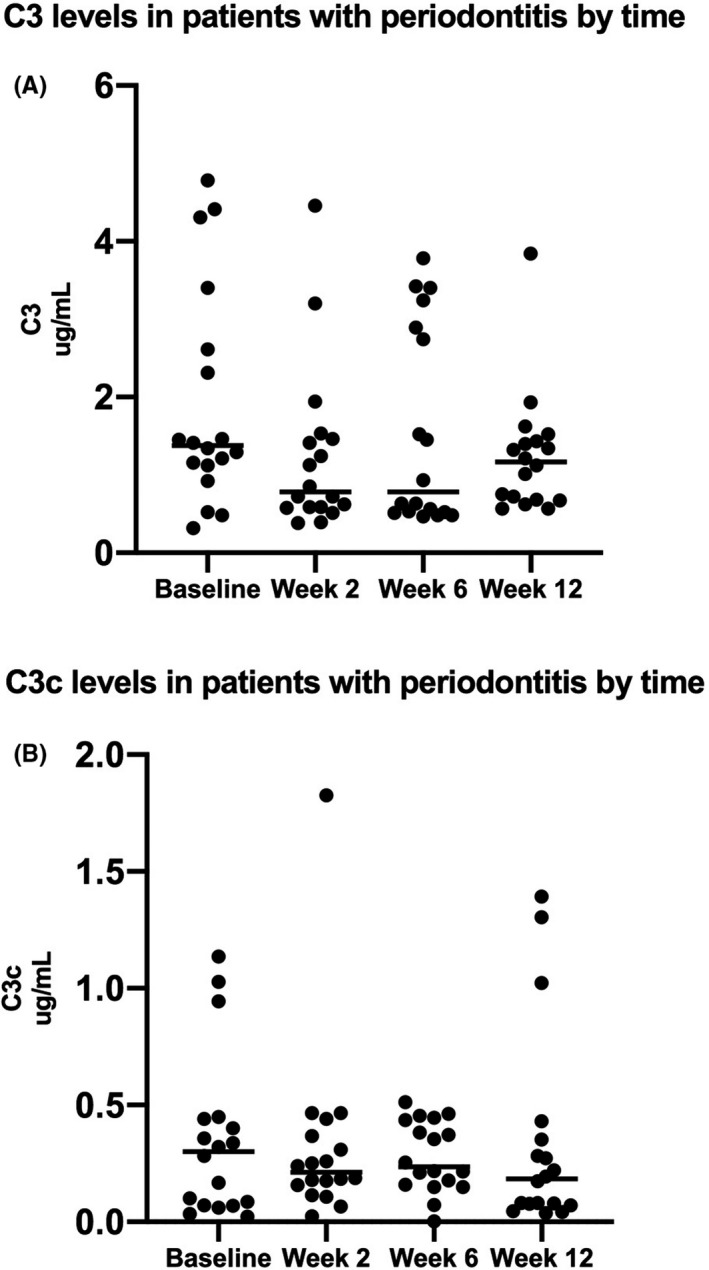
Pre‐ and post‐treatment levels of C3 and C3c in patients with periodontitis. The scatter dot‐plots illustrate salivary levels of (A) C3 (μg/mL) and (B) C3c (μg/mL) recorded at baseline and two‐, six‐, and twelve weeks post‐treatment in patients with periodontitis (n = 18). The horizontal lines show the median levels

The improved periodontal status was reflected by a decrease in both C3 and C3c levels at week two; however, the changes were not statistically significant (Figure 3).

### 3.3 Baseline salivary levels of C3 and C3c in good and poor responders

**Figure 4:**
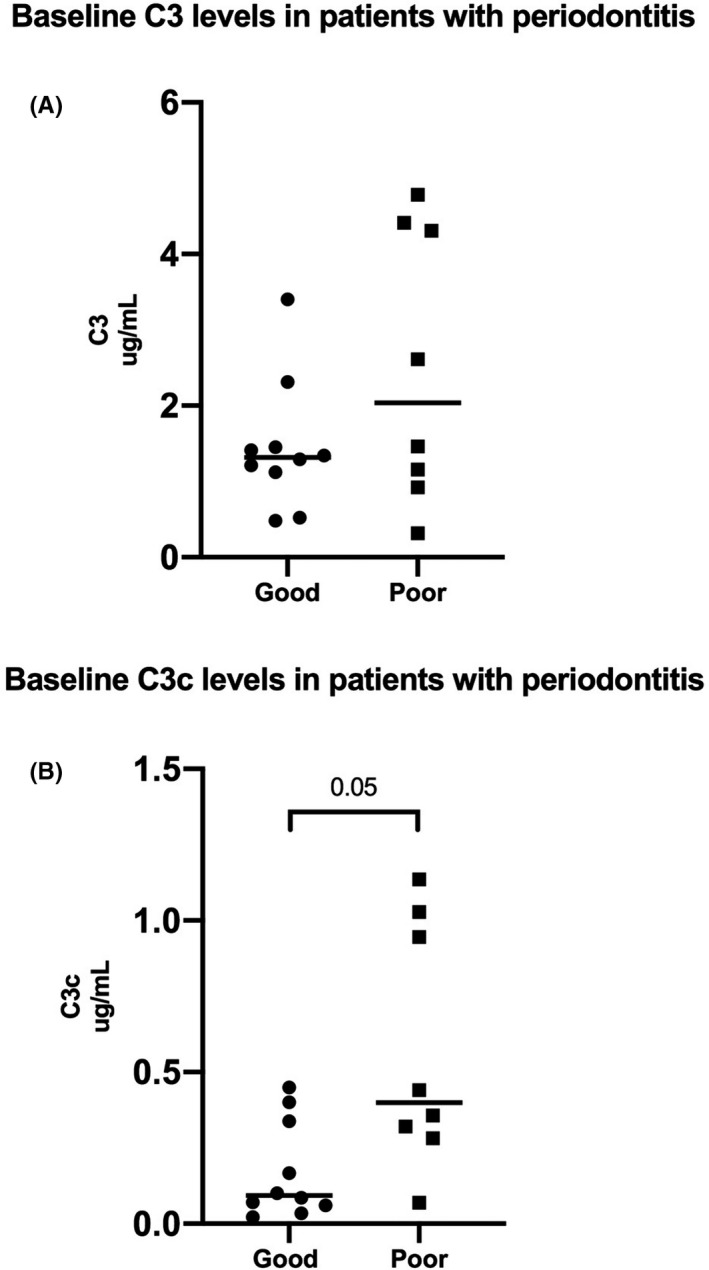
Baseline salivary C3 and C3c levels in good and poor responders to non‐surgical periodontal treatment. The scatter dot‐plots illustrate baseline saliva levels of (A) C3 (*P* > .05) and (B) C3c (*P* < .05) in good responders (n = 10) compared with poor responders (n = 8). The horizontal lines show the median levels in the two groups

Patients with periodontitis were grouped into good (n = 10) and poor responders (n = 8) based on a reduction in BOP above or below mean (mean level of the total BOP reductions from baseline to week 12 = 22%). Baseline values of total C3 did not differ significantly between the good and poor responders (Figure 4A). In contrast, the good responders had significantly lower baseline levels of C3c than the poor responders (*P* < .05), (Figure 4B).

**Figure 5:**
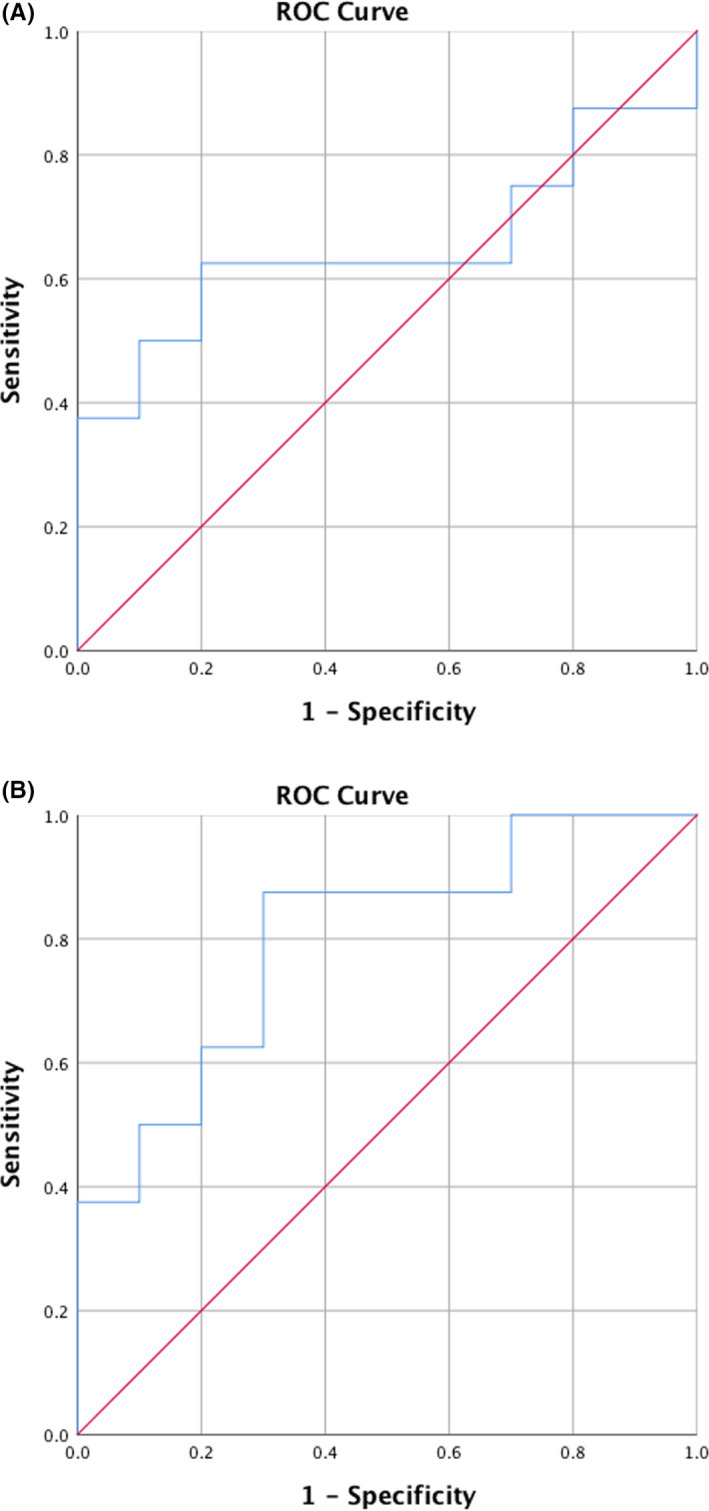
AUC for total C3 and C3c at baseline in identifying poor responders to non‐surgical periodontal treatment. ROC analysis of salivary baseline levels of A = C3 and B = C3c to discriminate between the good (n = 10) and the poor (n = 8) responders to periodontal treatment. (A) C3, AUC = 0.65, CI = [0.36;0.94], (*P* > .05) and (B) C3c, AUC = 0.80, CI = [0.59;1.00], (*P* < .05)

In line, baseline values of total salivary C3 could not distinguish the patients who responded poorly to treatment from patients who responded well (Figure 5A), but salivary baseline levels of C3c were able to discriminate between the two groups. Using a ROC analysis, an AUC = 0.80, CI = [0.59;1.00] was found for C3c (*P* < .05), (Figure 5B).

## 4 DISCUSSION

The aim of the study was to determine if measurements of salivary levels of total C3 and C3c had diagnostic and predictive value in regard to periodontitis and treatment thereof. Previously, salivary levels of total C3 and C4 have been associated with periodontitis.^30^ However, so far activation‐related complement split products may better reflect ongoing inflammation but have not been reported in saliva from patients with periodontitis. To our knowledge, this longitudinal study is the first to include measurements of salivary C3c split from the C3dg moiety in relation to periodontitis. We were able to do so by using an in‐house raised monoclonal antibody recognizing a neo‐epitope in C3c.^22^

Salivary levels of total C3 and its split product C3c were higher in patients with untreated periodontitis than in periodontally healthy controls. Moreover, both parameters were able to discriminate periodontitis from periodontal health. The increased salivary levels of C3 can be explained by both an inflammation‐related increase of vascular permeability and by an increase in local production of complement proteins by macrophages and dendritic cells.^31^ In line with our findings, increased levels of intact complement proteins have been related to gingival inflammatory activity and periodontitis.^16^, ^17^ For example, total C3 and C5 have been demonstrated present in gingival biopsies from patients with periodontitis but absent in biopsies from healthy individuals.^16^ Also, in gingival crevicular fluid (GCF) higher levels of total C3, C4, and C5 have been associated with inflamed periodontal tissues.^17^ Thus, beside a re‐demonstration of the association between intact complement proteins and periodontitis, our study shows a potential of split product C3c, as a marker of local and ongoing complement activation, and periodontitis.

In the present study, improvements of all clinical parameters were accompanied by decreases in salivary levels of total C3 and C3c levels. At week 2, a decrease in the inflammatory parameter BOP was reflected by decreases in levels of both total C3 and split product C3c. As in our study, associations of clinical improvements with complement proteins have been shown previously.^32^, ^33^ These findings, however, are based on assays using polyclonal antibodies, which cannot exclude detection of some intact C3, C3b, and iC3b fragments, which also contain the C3c moiety, in addition to isolated C3c. Nevertheless, they can indicate decreased complement levels, as a result of decreased vascular permeability and/or local production of C3, but not decreases in ongoing complement activation as in our assay. Finally, a downregulation of C3 genes has been demonstrated in gingival biopsies from patients recently treated for periodontitis compared with biopsies collected from periodontally healthy controls.^34^

The observed associations of C3 and C3c with periodontitis suggest that inhibition of these proteins can improve the treatment of the disease. In addition, recently performed studies in non‐human primates have demonstrated that C3 inhibition by Cp40 results in both decreased dysbiosis, inflammation, and osteoclastogenesis.^20^, ^35^, ^36^

Knowledge of the individual's disease progression and treatment response is important for optimizing the individual treatment plan for patients with periodontitis. The important finding of the present study was therefore that baseline values of C3c in saliva, to some degree, were able to identify the group of patients, who responded poorly to treatment and thereby had increased risk of further disease progression. This finding is pioneering in treatment of periodontitis but needs additional research in larger studies with a longer follow‐up period.

Some limitations apply to the present investigation, including the relatively small sample size. Inclusion of GCF (local) and plasma samples (systemic) would improve the biological understanding of the results. Furthermore, age differences between patients with periodontitis and periodontally healthy controls may have impact on our data, as GCF concentrations of the complement component C1 have previously been described to associate with age.^37^

It should be noted that C3c is not to a specific marker for periodontitis, but rather a marker indicating increased inflammatory activity. Thus, a recent study found an association of increased salivary levels of the split product C3c with presence of oral lichen planus, using the same monoclonal antibody as employed in the present study.^38^

In conclusion, high salivary baseline levels of complement component C3c were predictive of periodontitis and poor responses to treatment. C3c therefore has potential to diagnose periodontitis and predict treatment responses. In the future, clinical measurement of baseline C3c levels may contribute to risk assessment and monitoring of periodontitis, which may result in a better and more personalized treatment.

## CONFLICT OF INTEREST

All authors declare no conflict of interest.
